# Influence of gold nanoparticle surface chemistry and diameter upon Alzheimer’s disease amyloid-β protein aggregation

**DOI:** 10.1186/s13036-017-0047-6

**Published:** 2017-02-06

**Authors:** Kelly A. Moore, Kayla M. Pate, Deborah D. Soto-Ortega, Samuel Lohse, Nicholas van der Munnik, Mihyun Lim, Kaliah S. Jackson, Venetia D. Lyles, Lemeisha Jones, Nisha Glassgow, Vanessa M. Napumecheno, Shanee Mobley, Mark J. Uline, Rahina Mahtab, Catherine J. Murphy, Melissa A. Moss

**Affiliations:** 1 Biomedical Engineering Program, University of South Carolina, Columbia, SC 29208 USA; 2 Department of Chemical Engineering, University of South Carolina, 2C02 Swearingen Engineering Center, Columbia, SC 29208 USA; 3 Department of Biological Sciences, University of South Carolina, Columbia, SC 29208 USA; 4 Department of Biological and Physical Sciences, South Carolina State University, Orangeburg, SC 29117 USA; 5 Department of Chemistry, University of Illinois at Urbana-Champaign, Urbana, IL 61801 USA

**Keywords:** Alzheimer’s disease, Amyloid-β protein, Protein aggregation, Inhibition, Gold nanoparticles, Aggregate morphology

## Abstract

**Background:**

Deposits of aggregated amyloid-β protein (Aβ) are a pathological hallmark of Alzheimer’s disease (AD). Thus, one therapeutic strategy is to eliminate these deposits by halting Aβ aggregation. While a variety of possible aggregation inhibitors have been explored, only nanoparticles (NPs) exhibit promise at low substoichiometric ratios. With tunable size, shape, and surface properties, NPs present an ideal platform for rationally designed Aβ aggregation inhibitors. In this study, we characterized the inhibitory capabilities of gold nanospheres exhibiting different surface coatings and diameters.

**Results:**

Both NP diameter and surface chemistry were found to modulate the extent of aggregation, while NP electric charge influenced aggregate morphology. Notably, 8 nm and 18 nm poly(acrylic acid)-coated NPs abrogated Aβ aggregation at a substoichiometric ratio of 1:2,000,000. Theoretical calculations suggest that this low stoichiometry could arise from altered solution conditions near the NP surface. Specifically, local solution pH and charge density are congruent with conditions that influence aggregation.

**Conclusions:**

These findings demonstrate the potential of surface-coated gold nanospheres to serve as tunable therapeutic agents for the inhibition of Aβ aggregation. Insights gained into the physiochemical properties of effective NP inhibitors will inform future rational design of effective NP-based therapeutics for AD.

**Electronic supplementary material:**

The online version of this article (doi:10.1186/s13036-017-0047-6) contains supplementary material, which is available to authorized users.

## Background

In 1901, Alois Alzheimer examined a patient experiencing multiple neurological symptoms, including pronounced memory loss [1], marking the first diagnosis of what is now the most common neurodegenerative disorder, Alzheimer’s disease (AD). Amyloid plaques, comprised of aggregated amyloid-β (Aβ) protein [2] and found throughout the cerebral cortex [1], are a pathological hallmark of AD. While monomeric Aβ is inert [3], Aβ aggregates induce neurotoxicity [4], inhibit neuronal long-term potentiation [5–7], induce synapse loss [8], and disrupt memory and complex learned behavior [9]. As a result, halting Aβ aggregation is one promising therapeutic strategy for AD. However, extensive investigation of small molecules and peptides as inhibitors of Aβ aggregation has failed to yield a successful therapeutic, necessitating the exploration of novel therapeutic agents.

Nanoparticles (NPs) have emerged as attractive therapeutic and diagnostic tools with applications in medical imaging, analytics, and drug delivery [10–14]. NPs can be synthesized from a wide range of materials including metals, polymers, and carbon-based molecules [11–14]. Furthermore, the ease with which NP size, shape, and surface properties are controlled [10–14] render NPs an ideal tunable platform for therapeutic applications.

Among the growing body of potential therapeutic applications for NPs is their ability to modulate amyloid protein aggregation [15–19]. Inhibition of Aβ aggregation, specifically, has been reported for NPs ranging in size from <10 nm to several hundred nanometers and exhibiting diverse surface chemistries [20–28]. Moreover, these effects have been observed at picomolar NP concentrations and substoichiometric ratios of NP to protein. While a wide array of small molecules and peptides can disrupt Aβ aggregation [29, 30], none have been as effective as NPs at substoichiometric ratios, thus increasing their potential for delivery of therapeutically effective concentrations to the brain. However, variations in NP size and surface chemistry can result in the contrasting promotion of Aβ aggregation [21, 23, 24, 31–33]. Thus, there exists a need to better understand the impact of NP physiochemical properties upon Aβ aggregation.

Using spherical NPs that vary in surface coating and size, this study investigates the effect that NP surface chemistry, charge, and diameter have upon Aβ aggregation. Gold was selected as the NP core material because gold NPs are readily synthesized, easily functionalized, and highly stable against oxidative dissolution [34–36]. Examination of four NP surface chemistries as well as three different NP diameters revealed that electric charge, surface chemistry, and size all modulate the ability of gold nanospheres to inhibit Aβ aggregation. While NP diameter and surface chemistry impact the extent of inhibition, electric charge determines the ability to influence aggregate morphology. In particular, smaller, anionic NPs are superior inhibitors, halting aggregation at substoichiometric ratios as low as 1:2,000,000 with the protein. Theoretical calculations suggest that such low stoichiometry may be achieved through NP-induced alterations to local solution conditions, including pH and charge density. Together, these findings identify surface-coated gold NPs as potential therapeutic agents for AD and provide insight into the physiochemical properties displayed by NPs that effectively inhibit Aβ aggregation.

## Results and discussion

As part of the emergence of NPs in medical applications, development of NPs as inhibitors of amyloid protein aggregation has garnered attention [15–19]. With the ability to vary NP physiochemical properties, including material, size, and charge, NPs offer a tunable platform to modulate amyloid protein aggregation [37, 38]. However, the influence of NP characteristics on protein aggregation is poorly understood [15–17]. This study characterizes the inhibition of Aβ aggregation by gold nanospheres with varying surface chemistry and diameter. A cellular assay was used to probe the neurotoxicity of synthesized NPs, while a fluorescent amyloid-binding dye and transmission electron microscopy (TEM) were employed to characterize NP-induced alterations in Aβ aggregate formation and morphology, respectively. These investigations define a role for NP size, electric charge, and surface chemistry in determining inhibitory capabilities, and a theoretical model provides insight into the possible mechanism of inhibition.

### Toxicity of surface-coated gold NPs

**Fig. 1:**
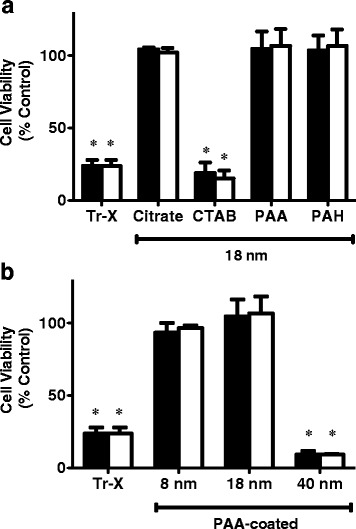
Toxicity of surface-coated gold NPs. SH-SY5Y human neuroblastoma cells were incubated (24 h) alone (*negative control*) or in the presence of 100 pM (*closed bars*) or 200 pM (*open bars*) NPs. **a** Treatment with 18 nm NPs coated with citrate, CTAB, PAA, or PAH. **b** Treatment with 8 nm, 18 nm, or 40 nm NPs coated with PAA. Treatment with 2% Triton-X (Tr-X) served as a positive control. Cellular viability was assessed using XTT reduction. Results are shown as the percentage of viable cells relative to the negative control and represent the mean of 2–3 independent experiments, performed with 6 replicates. Error bars represent SEM. *p < 0.001 vs. negative control

Toxicity of gold NPs is dependent upon their size, shape, and surface coating [39–43]. To probe the biocompatibility of synthesized NPs as therapeutic agents, their potential to elicit a toxic response was evaluated within human neuroblastoma SH-SY5Y cells, a widely used model for the study of prospective AD therapeutics. Following 24 h exposure of SH-SY5Y cells to surface-coated NPs, cellular metabolic activity was assessed using 2,3-bis(2-methoxy-4-nitro-5-sulfophenyl)-2H-tetrazolium-5-carboxanilide (XTT) reduction. At NP concentrations of 100 pM and 200 pM, cellular viability remained >95% following incubation with 18 nm NPs displaying citrate, poly(acrylic acid) (PAA), or polyelectrolytes poly(allylamine)hydrochloride (PAH) surface chemistries (Fig. 1a). In contrast, 18 nm cetyltrimethylammonium bromide (CTAB) coated NPs reduced cellular viability to less than 20%, a viability level comparable to cells treated with 2% Triton-X, thus indicating their propensity to induce toxicity. This result is congruent with observations that CTAB-coated nanoparticles are toxic toward other cell types [39–41], while their further overcoating reduces the toxic effects [39, 41]. When cells were incubated in the presence of PAA-coated NPs of varying size, NPs 8 nm and 18 nm in diameter did not elicit toxicity. In contrast, 40 nm PAA-coated NPs were toxic, with both 100 pM and 200 pM NPs reducing cellular viability to less than 10% (Fig. 1b). Similarly, other studies have shown size-dependent toxicity for nanoparticles, with larger sizes eliciting a more toxic response [42, 43]. Results here demonstrate that NP toxicity toward SH-SY5Y cells is influenced by both surface chemistry and size and that several NPs considered in the current study are inert in SH-SY5Y cultures.

### Effect of surface-coated gold NPs on ThT fluorescence detection of Aβ_1–40_ aggregates

**Fig. 2:**
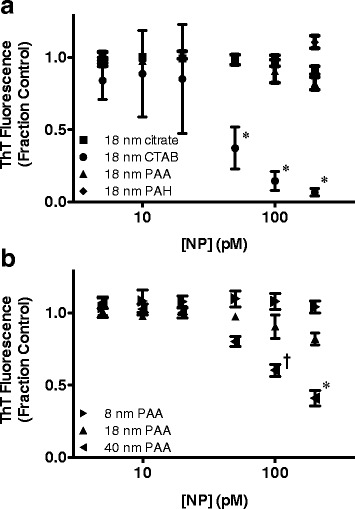
Effect of surface-coated gold NPs on ThT fluorescence detection of Aβ_1-40_ aggregates. Aβ_1–40_ fibrils diluted in 40 mM Tris–HCl (pH 8.0) to a final concentration of 5 μM were combined with 8.75 μM ThT and incubated (2 h) alone (control) or with 5 pM, 10 pM, 20 pM, 50 pM, 100 pM, or 200 pM NPs. **a** Incubation with 18 nm NPs displaying citrate (■), CTAB (●), PAA (▲), or PAH (♦) surface chemistries. **b** Incubation with 8 nm (▶), 18 nm (▲), or 40 nm (◀) NPs coated with PAA. ThT fluorescence was evaluated, and results are expressed as the fraction of ThT fluorescence observed for the control. Error bars represent SEM, *n* = 3. ^†^ *p* < 0.05 and *p < 0.001 vs. control

The plasmonic nature of NPs imparts an ability to absorb and scatter light, which may enhance or quench fluorescent signals [44]. This property presents the possibility that NPs could alter fluorescence of thioflavin T (ThT), which binds the β-sheet structure characteristic of fibrillar Aβ aggregates to yield a shifted, enhanced fluorescence and is thus commonly used to monitor the progression of aggregation. To ensure that observed differences in ThT fluorescence accurately reflect changes in Aβ aggregate formation, ThT fluorescence was assessed for 5 μM pre-formed fibrillar Aβ_1–40_ aggregates incubated for 2 h in the absence (control) or presence of 5–200 pM surface-coated NPs. These concentrations are representative of diluted solutions used for monitoring aggregation. Among 18 nm NPs, ThT fluorescence detection of Aβ_1-40_ fibrils was unaltered by NPs displaying citrate, PAA, and PAH surface chemistries (Fig. 2a). In contrast, CTAB-coated NPs significantly quenched ThT fluorescence detection of aggregated Aβ_1-40_ at concentrations of 50 pM and higher. When PAA-coated NPs of varying diameter were compared, neither 8 nm nor 18 nm NPs altered the detection of Aβ_1-40_ fibrils by ThT; however, ThT fluorescence detection was again significantly quenched by the presence of 40 nm PAA-coated NPs (Fig. 2b). Other studies have shown that small molecule inhibitors of Aβ aggregation can also reduce ThT fluorescence detection of Aβ aggregates, although via a different mechanism of binding competition [45, 46]. These studies advocate caution toward the unmitigated use of ThT fluorescence to study Aβ aggregation inhibitors. As a result, assessment of the effect of 18 nm CTAB-coated NPs and 40 nm PAA-coated NPs was limited to TEM analysis.

### 18 nm surface-coated gold NPs inhibit Aβ_1–40_ monomer aggregation and alter fibril morphology

**Fig. 3:**
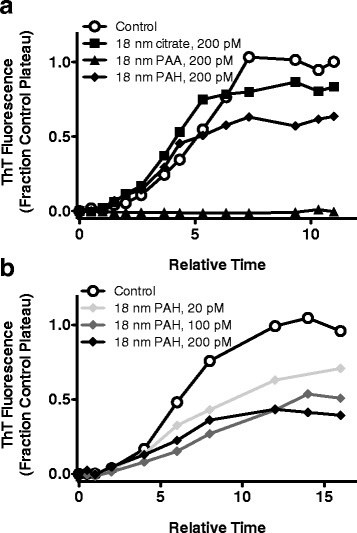
Effect of 18 nm surface-coated gold NPs on Aβ_1–40_ monomer aggregation. Aβ_1–40_ monomer diluted to 40 μM in 40 mM Tris–HCl (pH 8.0) was aggregated alone (control, Ο) or in the presence of surface-coated NPs. **a** Aggregation in the presence of 200 pM NPs displaying citrate (■), PAA (▲), or PAH (♦) surface chemistries. **b** Aggregation in the presence of 200 pM (♦), 100 pM (), or 20 pM () PAH-coated NPs. Monomer aggregation was induced by continuous agitation and monitored periodically via ThT fluorescence. ThT fluorescence, expressed as the fraction of the control equilibrium plateau, is plotted versus relative time, which is the fraction of the control lag time. Results are representative of 3–5 independent experiments

The effect of synthesized NPs on Aβ aggregation was evaluated using Aβ_1–40_, the most abundant monomeric isoform in vivo [47] as well as the dominant species found in amyloid plaques [48]. Aggregation of 40 μM monomeric protein was stimulated by continuous agitation, and ThT fluorescence was used to monitor the formation of β-sheet amyloid aggregates. Aβ_1–40_ aggregation yielded a characteristic growth pattern displaying an initial lag phase, indicative of nucleation, which was followed by rapid aggregate growth that ceased at equilibrium, as evidenced by plateau of the fluorescence signal (Fig. 3a). Inhibition of Aβ_1–40_ aggregation by NPs was quantified via changes in the lag time and equilibrium plateau. Extension of the lag time occurs early in aggregation and is indicative of an increased time to nucleation. Lag extension was calculated as a fold-change, relative to the control, in the time at which ThT fluorescence first increases. Reduction of the plateau fluorescence is evidenced at equilibrium and indicates a decrease in the total quantity of aggregates containing β-sheet structure. Plateau reduction was calculated as the percentage decrease in ThT fluorescence at equilibrium.

When monomer aggregation was stimulated in the presence of NPs, none of the NPs were capable of delaying nucleation to extend the lag time. However, at 200 pM several NPs did decrease the quantity of β-sheet aggregates formed at equilibrium (Fig. 3a). Aggregates formed in the presence of 200 pM citrate- or PAH-coated NPs exhibited a 19 ± 8% and 59 ± 9% reduction of the equilibrium plateau, respectively (Table 1). The most pronounced effect was observed with PAA-coated NPs, which completely abrogated aggregation. Inhibition of Aβ_1–40_ aggregation by surface-coated NPs was also observed to be dose dependent (Table 1). Inhibition by PAH-coated NPs decreased from nearly 60% at a concentration of 200 pM to less than 45% at a concentration of 20 pM (Fig. 3b, Table 1), and citrate-coated NPs were ineffective at concentrations below 200 pM (Table 1). PAA-coated NPs, however, continued to fully abrogate inhibition at concentrations as low as 20 pM, or a 1:2,000,000 substoichiometric ratio of NPs to Aβ_1-40_ (Table 1). To ensure these inhibitory effects were characteristic of the NPs and not just surface coatings, monomer aggregation was also performed in the presence of 100 μM solubilized sodium citrate, CTAB, PAA, or PAH; each failed to elicit any inhibitory effect (results not shown).

**Table 1:** Percent inhibition of Aβ_1–40_ monomer aggregation observed in the presence of surface-coated NPs^a,b^

NP type		[NP]		
Surface Modification	Diameter	20 pM	100 pM	200 pM
Citrate	18 nm	4.3 ± 2.6	0.0 ± 0.0	19 ± 8^*^
PAH	18 nm	45 ± 4^*^	47 ± 5^*^	59 ± 9^*^
PAA	18 nm	97 ± 2^*^	99 ± 0^*^	95 ± 3^*^
PAA	8 nm	95 ± 3^*^	92 ± 4^*^	94 ± 1^*^

*p < 0.001 compared to control

^a^Inhibition is expressed as plateau reduction, or the percentage of reduction in the equilibrium plateau compared to the control

^b^Parameters are expressed as mean ± SEM, *n* = 3–5

**Fig. 4:**
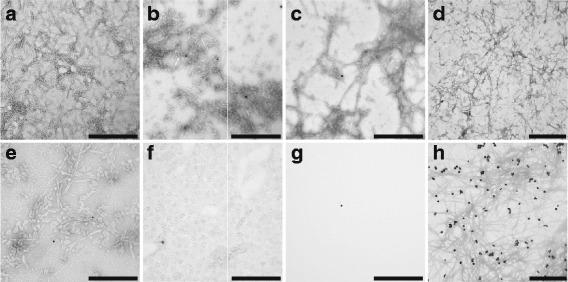
Effect of surface-coated gold NPs with different surface coatings and diameters on Aβ_1-40_ aggregate morphology. Aβ_1–40_ monomer diluted to 40 μM in 40 mM Tris–HCl (pH 8.0) was aggregated alone (control, panels **a**, **d**) or in the presence of 200 pM citrate-coated (panel **b**), PAH-coated (panel **c**), CTAB-coated (panel **e**), or PAA-coated (panel **g**) NPs 18 nm in diameter. Additionally, monomer was aggregated in the presence of 200 pM PAA-coated NPs exhibiting diameters of 8 nm (panel **f**) or 40 nm (panel **h**). Monomer aggregation was induced by continuous agitation, and the control reaction was monitored periodically via ThT fluorescence. Upon evidence of equilibrium, samples were gridded and visualized by TEM. Results are representative of 2 independent experiments. Images are shown relative to a scale bar of 500 nm at 100,000x (panels **a**-**c**, **e**-**g**) or 75,000x (panels **d**, **h**) magnification

To confirm inhibition of Aβ_1–40_ aggregation by synthesized NPs as well as to further investigate changes in aggregate morphology, TEM images were acquired following a time point after which aggregation reactions had reached equilibrium. Aggregates formed in the absence of NPs exhibit a network of filamentous fibrils comprised of single or multiple strands (Fig. 4a, d). This morphology is in agreement with other studies of Aβ aggregation [20, 24, 27, 33].

When Aβ_1-40_ aggregates were formed in the presence of 200 pM citrate-coated (Fig. 4b) or PAH-coated (Fig. 4c) NPs, the quantity of aggregates was reduced compared to the control. These results corroborate the plateau reductions observed via ThT fluorescence. TEM additionally facilitated examination of the influence of CTAB-coated NPs on Aβ_1–40_ aggregation, for which analysis by ThT fluorescence was precluded. Aggregates formed in the presence of 200 pM CTAB-coated NPs exhibited a reduction in aggregate quantity (Fig. 4e), demonstrating that these NPs can also inhibit Aβ_1–40_ aggregation. When Aβ_1–40_ aggregation was stimulated in the presence of 200 pM 18 nm PAA-coated NPs, an absence of filamentous aggregates was observed (Fig. 4g), substantiating the ability of these NPs to abrogate aggregation. Together, TEM results confirm the relative inhibitory capabilities of surface-coated NPs observed by ThT fluorescence as well as the complete inhibition imparted by PAA-coated NPs.

While the most effective inhibitors were NPs coated with anionic PAA, NPs exhibiting both negative and positive surface charges inhibited Aβ aggregation. This observation agrees with previous studies that have described both anionic and cationic NPs as inhibitors of protein aggregation [20, 21, 23–25, 28, 49–55]. Among the NPs examined within the current study, however, those with anionic citrate and PAA coatings were more effective inhibitors than those with cationic CTAB and PAH coatings. This finding aligns with other studies that have observed superior inhibitory capabilities by anionic NPs over cationic NPs [20, 21, 23, 25, 28]. Among anionic NPs, PAA-coated particles exhibited superior inhibitory capabilities over citrate-coated particles. Other studies also report variances in inhibition of amyloid protein aggregation by NPs displaying different surface chemistries with the same electric charge [22, 25]. In comparison to monomeric citrate, polymeric PAA will exhibit molecular reorganization transitions with the local solution environment [56]. These transitions can result in spatially dependent changes to physical parameters that may facilitate inhibition, as discussed in the next section.

TEM images further revealed that NPs with different coatings exert different effects on aggregate morphology. While aggregates formed in the presence of anionic citrate-coated NPs exhibited a morphology similar to the control (Fig. 4b), aggregates formed in the presence of cationic NPs demonstrated altered morphologies. PAH-coated NPs induced the formation of an increased number of thin, elongated aggregate structures (Fig. 4c), while CTAB-coated NPs induced the formation of short, thick associated aggregates (Fig. 4e). Thus, these results demonstrate the ability of cationic, but not anionic, NPs to influence aggregate morphology. Other studies have described similar NP-induced changes in aggregate structure, with a diverse array of morphologies reported [20, 21, 26, 49, 50, 52, 54, 55]. However, these observations are not confined to cationic NPs.

### NP size influences inhibition of Aβ_1–40_ monomer aggregation by PAA-coated gold NPs

**Fig. 5:**
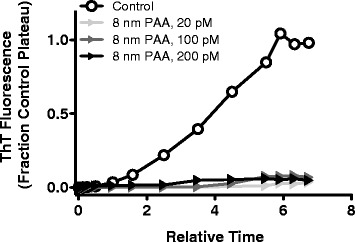
Effect of 8 nm PAA-coated gold NPs on Aβ_1–40_ monomer aggregation. Aβ_1-40_ monomer diluted to 40 μM in 40 mM Tris–HCl (pH 8.0) was aggregated alone (control, Ο) or in the presence of 8 nm PAA-coated NPs at concentrations of 200 pM (▶), 100 pM (), or 20 pM (). Monomer aggregation was induced by continuous agitation and monitored periodically via ThT fluorescence. ThT fluorescence and time values are presented as in Fig. 3. Results are representative of 3 independent experiments

The complete inhibition of Aβ_1–40_ aggregation observed in the presence of 18 nm PAA-coated NPs prompted further experimentation to elucidate the effect of PAA-coated NP size on inhibitory capabilities. PAA-coated NPs exhibiting diameters of 8 nm, 18 nm, and 40 nm were examined for their ability to attenuate Aβ_1–40_ aggregation. These NP sizes were selected to span the range of sizes able to cross the blood–brain barrier and undergo clearance from the body [57, 58]. Similar to the inhibition observed in the presence of 18 nm PAA-coated NPs, the smaller 8 nm NPs reduced the equilibrium plateau by >90% at concentrations as low as 20 pM (Fig. 5, Table 1), demonstrating their effectiveness as Aβ aggregation inhibitors. This result was corroborated by the absence of aggregate material observed via TEM (Fig. 4f). In contrast, 40 nm PAA-coated NPs were ineffective at preventing the formation of Aβ_1-40_ aggregates. Although these NPs quenched ThT fluorescence (Fig. 2), precluding their assessment via ThT, their inability to prevent aggregate formation was evidenced by TEM images displaying a similar quantity of aggregate material (Fig. 4h) compared to the control of equivalent magnification (Fig. 4d). These results demonstrate that only smaller PAA-coated NPs are capable of serving as effective inhibitors of Aβ_1–40_ aggregation. This observation is in agreement with other studies reporting variations in inhibition of amyloid protein aggregation by NPs exhibiting different sizes [21, 28]. As in the current study, larger NPs are consistently less effective inhibitors, suggesting that high NP curvature may be needed to impart inhibitory capabilities.

Both 8 nm and 18 nm PAA-coated nanospheres were capable of abrogating Aβ aggregation at a substoichiometric ratio of 1:2,000,000. While other studies have proposed NP-protein binding as the mode of aggregation inhibition, this extremely low NP to monomer ratio suggests that these surface-coated NPs are acting via another mechanism. Moreover, TEM images show that NPs did not co-localize with aggregates and that morphological changes were not isolated to regions near NPs. A similar lack of interaction with aggregated protein is also reported for other NP types [20, 50, 54, 59]. These observations suggest a dynamic interaction between NP-localized protein and the bulk solution.

### Theoretical calculations indicate that surface charged NPs alter local solution conditions that can influence Aβ aggregation

The strikingly low stoichiometry at which inhibition of Aβ_1–40_ aggregation by PAA-coated NPs was observed as well as the lack of association between NPs and Aβ aggregates within TEM images suggest that interactions other than sequestration of Aβ_1–40_ monomer at the NP surface may play a role in the inhibitory capabilities of surface-coated NPs. A potential mechanism that can account for these congruent observations exists in the effect that the curved, charged NP surface has upon local solution conditions, including pH and ionic strength [21], both of which significantly influence Aβ aggregation [60–62]. Specifically, Aβ aggregation is attenuated in the presence of acidic and basic pH [61, 63–65], while aggregation is promoted by the presence of a higher ionic strength, or charge density [62, 66–70]. Moreover, both solution pH [60, 65, 66, 71] and ionic strength [66, 67, 70] can modulate aggregate morphology. To explore this possibility, a self-consistent molecular field theory (SCMFT) was developed and implemented to describe molecular organization near the curved, charged NP surface. Theoretical calculation of the equilibrium concentrations of solution species allowed for the determination of local solution pH and charge density.

**Fig. 6:**
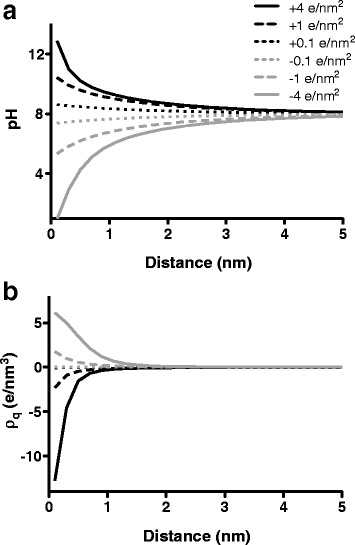
Effect of NP surface charge on local solution pH and charge density. The equilibrium molecular organization near the surface of NPs was modeled using a SCMFT incorporating water, hydronium, hydroxide, chloride, and Tris. Theoretical calculations were performed for 18 nm NPs in the presence of 40 mM Tris–HCl (pH 8.0) for NPs exhibiting a positive (*black lines*) or negative (*grey lines*) surface charge with absolute surface charge (σ_q_) magnitudes of 4 e/nm^2^ (*solid lines*), 1 e/nm^2^ (*dashed lines*), or 0.1 e/nm^2^ (*dotted lines*). Local solution pH (panel **a**) and charge density (ρ_q_) (panel **b**) are plotted as a function of distance from the NP surface

Theoretical calculations demonstrate pronounced changes in solution pH and solution charge density near the surface of 18 nm NPs suspended in 40 mM Tris–HCl (pH 8.0) (Fig. 6). Cationic NP surfaces induce an elevated local solution pH and a negative charge density, while anionic NP surfaces depress the local solution pH and induce a positive solution charge density. These changes result from the system localizing counterions near the NP surface to balance the NP surface charge. The magnitude of NP-induced alterations is consistent with conditions under which altered Aβ aggregation would occur [37, 61, 62]. Moreover, a greater absolute magnitude of surface charge produces more pronounced changes in the local solution molecular organization, leading to larger deviations from the bulk solution. This observation is congruent with prior experimental reports in which NPs with a higher absolute magnitude of surface charge imparted greater inhibition [22, 25]. For NPs with a large magnitude of surface charge, these effects extend several nanometers beyond the NP surface. This spatial pervasiveness would allow protein to exchange between local and bulk solution conditions. Such exchange may impede the formation of organized amyloid structures or disrupt those structures formed within the bulk solution. Previous observations support such a dynamic exchange [25, 59, 72] along with the dominance of nanoscale surface properties over the bulk solution [16], which may be partly due to the persistence of protein structures formed near the NP surface [72].

Interestingly, an asymmetry exists about the bulk solution values, with cationic NPs eliciting a more pronounced change in charge density. This asymmetry is a manifestation of differences between counterions that become localized to balance the NP surface charge. The cationic NP surface draws hydroxide and chloride to its surface, with chloride being the predominant species, while the anionic NP surface draws hydronium and Tris to its surface, with Tris predominating. In the latter case, because Tris is in chemical equilibrium with its local environment through the acid dissociation reaction (*tr* ^+^ ⇄ *tr* + *H* ^+^), the fraction of Tris that is charged, and hence capable of balancing the anionic NP surface charge, is interdependent upon the local pH environment, thus causing a distinct effect upon molecular organization. This asymmetric magnitude in charge density combined with the opposing changes in both local solution pH and charge density induced by anionic vs. cationic NPs may contribute to the distinct inhibitory effects observed for anionic and cationic NPs, including the stronger inhibitory capabilities of anionic NPs and the ability of cationic, but not anionic, NPs to alter aggregate morphology. Moreover, polymeric PAA can produce steric hindrance that results in a preference for localizing protons over bulky ions, such as Tris. The result is a more pronounced effect on the local pH compared to citrate, congruent with the enhanced effectiveness of PAA-coated vs. citrate-coated NPs.

Overall, theoretical results parallel experimental observations in both the current and prior studies, supporting the hypothesis that inhibition of amyloid aggregation may stem, in part, from NP-induced changes in local solution conditions.

## Conclusion

This study provides evidence that electric charge, surface chemistry, and size can modulate the ability of gold nanospheres to inhibit Aβ aggregation. NP surface chemistry and size influence the extent of inhibition, while electric charge defines NP ability to alter aggregate morphology. Overall, PAA-coated NPs 18 nm and smaller are superior inhibitors, abrogating aggregation at substoichiometric ratios as low as 1:2,000,000 with Aβ. Such low stoichiometric ratios coupled with the lack of NP-aggregate association prompted investigation for NP-induced changes in local solution conditions to influence aggregation. A theoretical model describing changes in local solution pH and charge density displays congruencies with experimental observations to support this potential mechanism. Cell viability assays further demonstrated that the most effective NP inhibitors are non-toxic. Together, these findings identify surface-coated gold nanospheres as potential tunable therapeutic agents for the inhibition of Aβ aggregation and provide insight into the physiochemical properties of effective NP inhibitors.

## Methods

### Materials

Aβ_1–40_ was purchased from AnaSpec, Inc. (San Jose, CA). Gold (III) trichloride hydrate HAuCl_4_ · 3H_2_O, trisodium citrate, sodium borohydride (NaBH_4_), ascorbic acid, CTAB, ThT, Triton-X 100, XTT, and all cell culture media and reagents were purchased from Sigma-Aldrich (St. Louis, MO). The polyelectrolytes PAH and PAA were also obtained from Sigma-Aldrich and used without further purification. Sodium chloride (NaCl) was purchased from Fisher Scientific. Uranyl acetate was purchased from Electron Microscopy Sciences (Hatfield, PA).

### Surface-coated gold NP synthesis and characterization

Surface-coated gold NPs of average core diameters 8 nm, 18 nm, and 40 nm were synthesized using a previously reported seeded growth method [73], described in detail within Additional file 1. NPs in their citrate-capped form were either used for experimentation, coated with CTAB, which forms a bilayer on the surface causing the trimethylammonuim headgroup to face the aqueous solvent, or electrostatically over-coated in a layer-by-layer fashion with PAA and PAH [74]. Therefore, at pH 7, the nanomaterials would present either a cationic (CTAB and PAH) or anionic (citrate and PAA) surface. NPs were characterized using TEM and UV–vis absorbance spectroscopy.

### Toxicity of surface-coated gold NPs

Potential toxicity of surface-coated NPs was probed in human neuroblastoma SH-SY5Y cells (American Type Culture Collection, Manassus, VA). Cellular reduction of XTT was employed to evaluate cellular metabolic activity following NP exposure. Cells, sustained and prepared for experiments as described in Additional file 1, were incubated (24 h) with NPs (100 pM or 200 pM) diluted into medium, with medium alone (negative control), or with 2% Triton-X 100 in medium (positive control). Following incubation, cells were washed and treated (24 h) with 0.33 mg/mL XTT and 8.3 μmol/L phenozene methyl sulfate. Metabolically active cells reduce XTT to an orange formazan product, for which absorbance (450 nm) was measured using a BioTek Synergy 2 microplate reader (Winooski, VT). Results are reported as a percentage of the negative control following background (medium containing XTT) subtraction.

### Aβ_1–40_ monomer aggregation

Aβ_1–40_ monomer aggregation assays were performed similar to previously described methods [75]. Briefly, Aβ_1–40_ monomer, purified via size exclusion chromatography (SEC) as described in Additional file 1, was diluted to 40 μM in 40 mM Tris–HCl (pH 8.0) and agitated (vortex, 800 rpm, 25 °C) alone (control) or with 20-200 pM NPs. Periodically, a 20 μL aliquot was removed and combined with 140 μL of 10 μM ThT, an amyloid-binding dye that yields a shifted, enhanced florescence upon recognition of the characteristic β-sheet structure of fibrillar Aβ aggregates. Fluorescence (excitation = 450 nm, emission = 470-500 nm) was evaluated using a Perkin-Elmer LS-45 luminescence spectrometer (Waltham, MA). Fluorescence values were calculated as the integrated area under the emission curve with baseline (ThT alone) subtraction and plotted vs. aggregation time.

### ThT detection of Aβ_1–40_ aggregates in the presence of surface-coated gold NPs

To ensure that NPs do not compromise ThT detection, ThT fluorescence was evaluated for 5 μM Aβ_1–40_ pre-formed fibrils, prepared as described in Additional file 1, in the presence of 8.75 μM ThT and 5-200 pM NPs (concentrations congruent with those of diluted samples used to monitor aggregation). ThT fluorescence was measured after 2 h incubation. Compromised ThT detection was evaluated as a decrease in ThT fluorescence relative to that observed for fibrils in the absence of NPs and expressed as the fraction of ThT fluorescence observed for the control.

### Morphological evaluation of Aβ_1–40_ aggregates

To evaluate Aβ_1–40_ aggregate morphology, monomer aggregation reactions were prepared for TEM following the time point at which the control reaction reached equilibrium (assessed via ThT). As described previously [75], a 10 μL sample was placed on a 300 mesh formvar-carbon supported copper grid (Electron Microscopy Sciences, Hatfield, PA). After 3 min, the sample was wicked away from the bottom side of the grid using filter paper. Sample application was repeated twice, and grids were allowed to air dry (24 h). Gridded samples were stained (10 min) with 2% aqueous uranyl acetate, excess stain was wicked away, and grids were allowed to dry (24 h). Imaging was performed using a JEOL 200CX TEM (Tokyo, Japan) with an accelerating voltage of 120 kV. Blinded observation of samples with random selection of grid areas was implemented to reduce bias.

### Statistical analysis

Using GraphPad Prism 5 software (San Diego, CA), the effect of NPs on aggregation was evaluated using a one-way analysis of variance (ANOVA) with Dunnett’s post-test, and the effect of NPs on ThT detection was evaluated using a two-way ANOVA with Bonferroni post-test. *p* < 0.05 was considered significant.

### Thermodynamic model

A SCMFT was developed and parameterized to model the equilibrium molecular organization near the interface of surface-coated NPs suspended in 40 mM Tris–HCl (pH 8.0). The NP surface was modeled as a sphere bearing a fixed surface charge where spherical symmetry was imposed. Five mobile species (water, hydronium, hydroxide, chloride, and Tris) were accounted for explicitly to capture the solvent environment. The chemical equilibrium of Tris is integrated into the model and made dependent upon the local solvent environment. The concentration of chloride was parameterized to achieve electroneutrality within the bulk solution. The SCMFT is expressed mathematically as a dimensionless free energy functional. The equilibrium molecular organization is determined through the minimization of this functional. Further details can be found in Additional file 1. This model was used to predict the molecular organization near the NP surface, from which the pH and charge density were determined a function of distance from the NP surface. Calculations were performed for 18 nm NPs with surface charges ranging from 4 to −4 e/nm^2^.

## Additional file

Additional file 1: Supporting Information. (DOCX 42 kb)

## Abbreviations

AD: Alzheimer’s disease
ANOVA: Analysis of variance
Aβ: Amyloid-β protein
BSA: Bovine serum albumin
CTAB: Cetyltrimethylammonium bromide
NP: Nanoparticle
PAA: Poly(acrylic acid)
PAH: Poly(allylamine)hydrochloride
SCMFT: Self-consistent molecular field theory
SEC: Size exclusion chromatography
TEM: Transmission electron microscopy
ThT: Thioflavin T
XTT: 2,3-bis(2-methoxy-4-nitro-5-sulfophenyl)-2H-tetrazolium-5-carboxanilide
