# Supplementary material for: Influence of gold nanoparticle surface chemistry and diameter upon Alzheimer’s disease amyloid-β protein aggregation
Source: J Biol Eng. 2017 Feb 6;11:5. doi: 10.1186/s13036-017-0047-6 (PMC5292815; doi:10.1186/s13036-017-0047-6)
Supplement: Additional file 1: — Supporting Information. (DOCX 42 kb) [file 13036_2017_47_MOESM1_ESM.docx]

**Supporting Information**

**Gold Nanoparticle (NP) Synthesis and Characterization**

Using glassware cleaned by aqua regia and rinsed with deionized water prior to use, NP seeds (3.5 nm) were synthesized by preparing a solution containing 2.5x10^-4^ M gold tetrachloroaurate (HAuCl_4_) and 2.5x10^-4^ M trisodium citrate in nanopure deionized water. To this solution, 0.6 mL of ice-cold 0.01 M sodium borohydride was added, with vigorous stirring. Following sodium borohydride addition, the solution turned red‑orange, indicating the formation of small NPs. Next, the NP solution was heated and boiled with 1% sodium citrate solution for 10 min in order to create citrate-coated gold NPs. These particles were stirred for 2 h, and aliquots were either purified by centrifugation and washing for use in experimentation or used unpurified as the seed solution for synthesis of larger gold NPs.

Subsequent growth solutions were prepared using a 200 mL solution containing 2.5x10^‑4^ M HAuCl_4_ and 0.08 M cetyltrimethylammonium bromide (CTAB). Aliquots (7.5 mL) of this solution were combined with 55 µL of ascorbic acid thereby reducing Au(III) to Au(I), turning the growth solution clear and colorless. Successively larger diameter CTAB‑stabilized NPs were then prepared by transferring 1 mL aliquots of the seed solution to the growth solutions. NP growth occurred over the course of several hours, after which NPs were purified by centrifugation and washing. NPs were either coated with CTAB or electrostatically over-coated in a layer-by-layer fashion with poly(acrylic acid) (PAA) and poly(allylamine)hydrochloride (PAH) [1]. Following polymer wrapping, NPs were again purified by centrifugation and washing.

NP size was characterized by transmission electron microscopy (TEM) analysis. Here, purified NP solutions were drop-cast onto formvar-coated Cu/SiO grids (Ted Pella, Redding, CA) and imaged using a JEOL 2100 TEM (Tokyo, Japan). Purified NP solution concentrations were determined by UV-vis absorbance spectroscopy using a Cary 500 UV-vis-NIR spectrophotometer (Agilent Technologies, Santa Clara, CA).

**Cell Culture**

Human neuroblastoma SH-SY5Y cells were sustained in a 1:1 mixture of Ham’s F12K medium and DMEM (F12K/DMEM) containing 10% fetal bovine serum (FBS), 1000 units/mL penicillin, and 100 µg/mL streptomycin. Cells were seeded (5x10^4^ cells/well) onto clear-sided 96‑well tissue culture plates (VWR, Radnor, PA) 24 h prior to treatment and sustained (37 °C, 5% CO_2_) in F12K/DMEM medium containing 1% FBS, 1000 units/mL penicillin, and 100 µg/mL streptomycin. All incubations were performed at 37 °C with 5% CO_2_.

**Purification of Aβ_1-40_ Monomer**

Lyophilized Aβ_1-40_ peptide was stored desiccated at -20 °C. As described previously [2], the peptide was reconstituted to 2 mg/mL in 50 mM NaOH to dissociate preformed small aggregates. Monomeric protein was resolved from any remaining aggregates via size exclusion chromatography (SEC) on a Superdex 75 10/300 GL column (GE Healthcare, Piscataway, NJ). Prior to purification, the column was equilibrated in 40 mM Tris-HCl (pH 8.0) and pre-treated with 2 mg/mL bovine serum albumin (EMD Biosciences, San Diego, CA) to minimize non-specific interactions between the matrix and the protein. Eluted Aβ_1‑40_ monomer concentrations were determined using UV-vis spectrometry (λ=276 nm, ε=1450 M^‑1^cm^‑1^) [3]. Purified monomeric Aβ_1-40_ was used fresh or stored at 4 °C for no longer than 5 days.

**Preparation of Aβ_1-40_ Fibrils**

Aβ_1-40_ fibrils were prepared from SEC isolated monomer. As described previously [2], 60 µM monomeric Aβ_1-40_ in 40 mM Tris-HCl (pH 8.0) was agitated (25 °C, 24 h) in the presence of 250 mM NaCl. Fibrils were separated from soluble Aβ_1-40_ species via centrifugation (13,000x*g*, 10 min). Supernatant, containing monomer and soluble aggregates, was removed, and the pellet was resuspended in 40 mM Tris-HCl (pH 8.0). Fibril concentrations were calculated from the fraction of pelleted protein. Fibrils were stored at 4 °C for no longer than 7 days.

**Thermodynamic Model**

For the physical situation, in which the volume, temperature, and chemical potential of all species is fixed, the thermodynamic potential minimized at equilibrium is the Grand potential [4]. For a NP of radius $R$, the dimensionless free energy functional per area is given by:

|  | $w=\frac{\beta\Omega}{A\left( R \right)}=\sum_{i=\{w,{Cl}^{-},tr\}} \int G\left( r \right)\rho_{i}\left( r \right)\left( \ln\rho_{i}\left( r \right)v_{w}-1 \right)dr$ | | (1) |
| --- | --- | --- | --- |
|  |  | $+\sum_{i=\{H^{+},{OH}^{-}\}} \int G\left( r \right)\rho_{i}\left( r \right)\left( \ln\rho_{i}\left( r \right)v_{w}-1+\beta\mu_{i}^{o} \right)dr$  $+\beta\int G\left( r \right)\left[ \rho_{q}\left( r \right)\psi\left( r \right)-\frac{1}{2}\varepsilon_{o}\varepsilon\left( \nabla_{r}\psi\left( r \right) \right)^{2} \right]dr+\beta\sigma_{q}\psi\left( R \right)$  $+\int G\left( r \right)\rho_{tr}\left( r \right)\left[ f\left( r \right)\left( \ln f\left( r \right)+\beta\mu_{tr+}^{o} \right)+\left( 1-f\left( r \right) \right)\left( \ln\left( 1-f\left( r \right) \right)+\beta\mu_{{tr}^{o}}^{o} \right) \right]dr$  $-\sum_{i=\left\{ w,{OH}^{-},{Cl}^{-},tr \right\}} \beta\mu_{i}\int G\left( r \right)\rho_{i}\left( r \right)dr-\beta\mu_{H^{+}}\int G\left( r \right)\left( \rho_{H^{+}}\left( r \right)+f\left( r \right)\rho_{tr}\left( r \right) \right)dr$  $+\beta\int G\left( r \right)\pi\left( r \right)\left( \phi_{w}\left( r \right)+\phi_{H^{+}}\left( r \right)+\phi_{{OH}^{-}}\left( r \right)+\phi_{{Cl}^{-}}\left( r \right)+\phi_{tr}\left( r \right)-1 \right)dr$ |  |

The first and second terms account for the mixing entropy of the five mobile species, where $\rho_{i}\left( r \right)$ is the density of species $i$ at radial position $r$. We adopt the convention that $v_{i}$ is the volume of species $i$ with water being represented by the index $w$. $\mu_{i}^{o}$ is the standard state chemical potential of species $i$. $G\left( r \right)$ is defined as a geometrical factor describing the change in available volume as a function of the radial coordinate [5]. In the spherical coordinate system, $G\left( r \right)=\left( r/R \right)^{2}$.

The third term accounts for electrostatic energy, where$\beta$ is the inverse temperature ($1/{k_{B}T}$), $\psi\left( r \right)$ is the electrostatic potential, $\varepsilon_{o}$ is the permittivity of free space, and $\varepsilon$ is the relative dielectric constant. $\rho_{q}\left( r \right)$ is the overall charge density at $r$ and is defined as:

|  | $\rho_{q}\left( r \right)=\rho_{H^{+}}\left( r \right)q_{H^{+}}+\rho_{{OH}^{-}}\left( r \right)q_{{OH}^{-}}+\rho_{{Cl}^{-}}\left( r \right)q_{{Cl}^{-}}+f\left( r \right)\rho_{tr}\left( r \right)q_{{tr}^{+}}$ | (2) |
| --- | --- | --- |
|  |  |  |

where $q_{i}$ is the charge of species $i$ and $f\left( r \right)$ is the fraction of charged Tris molecules at $r$. The fourth term in the functional accounts for the contribution to the free energy arising from the charged surface of the NP. Here, $\psi\left( R \right)$ is the electrostatic potential at the NP surface where $r\equiv R$.

The fifth term in the free energy functional accounts for the free energy arising from the chemical equilibrium of Tris [6]. Note that $\rho_{tr}\left( r \right)$ denotes the density of all Tris molecules at $r$, both protonated and neutral, whereas $\mu_{tr+}^{o}$ and $\mu_{{tr}^{o}}^{o}$ represent the standard state chemical potentials of Tris specifically in the protonated and neutral states, respectively.

Terms six and seven in the free energy functional account for the chemical potentials of all species. The final term serves to enforce incompressibility through the LaGrange multiplier,$\pi\left( r \right)$. $\phi_{i}\left( r \right)$ denotes the volume fraction of the indicated species at $r$. The physical interpretation of these$\pi\left( r \right)$ terms is the osmotic pressure at that position.

The thermodynamic potential of Eq 1 is that which is minimized at equilibrium. Taking the functional derivative with respect to variables of interest and setting the resulting expression equal to zero yields the equilibrium expression for those variables [7]. Using this method, the equilibrium expressions for the densities of all mobile species are derived:

|  | $\rho_{w}\left( r \right)v_{w}=exp\left( -\beta\pi\left( r \right)v_{w} \right)$  $\rho_{H^{+}}\left( r \right)v_{w}=exp\left( \beta\mu_{H^{+}}-\beta\mu_{H^{+}}^{o}-\beta\pi\left( r \right)v_{H^{+}}-\beta\psi\left( r \right)q_{H^{+}} \right)$  $\rho_{{OH}^{-}}\left( r \right)v_{w}=exp\left( \beta\mu_{{OH}^{-}}-\beta\mu_{{OH}^{-}}^{o}-\beta\pi\left( r \right)v_{{OH}^{-}}-\beta\psi\left( r \right)q_{{OH}^{-}} \right)$  $\rho_{{Cl}^{-}}\left( r \right)v_{w}=exp\left( \beta\mu_{{Cl}^{-}}-\beta\pi\left( r \right)v_{{Cl}^{-}}-\beta\psi\left( r \right)q_{{Cl}^{-}} \right)$  $\rho_{tr}\left( r \right)v_{w}=\frac{1}{1-f\left( r \right)}exp\left( \beta\mu_{tr}-\beta\mu_{{tr}^{+}}^{o}-\beta\pi\left( r \right)v_{tr} \right)$ | (3) |
| --- | --- | --- |

Likewise, the local equilibrium fraction of charged Tris is given by:

|  | $f\left( r \right)=\frac{1}{1+\frac{K_{a}^{o}\phi_{w}\left( r \right)}{\phi_{H^{+}}\left( r \right)}}$ | (4) |
| --- | --- | --- |

Note that the expression for $f\left( r \right)$ is obtained after mathematical manipulation. $K_{a}^{o}$ denotes the standard state equilibrium constant for the acid dissociation reaction of Tris and is defined as $K_{a}^{o}=exp[-\beta(\mu_{tr}^{o}+\mu_{H^{+}}^{o}-\mu_{{tr}^{+}}^{o})]$.

Extremization of the free energy functional with respect to the electrostatic potential yields the familiar Poisson equation and boundary conditions for this physical situation:

|  | $\nabla_{r}^{2}\psi\left( r \right)=-\frac{\rho_{q}\left( r \right)}{\varepsilon_{o}\varepsilon}$ | $\left. \frac{d\psi\left( r \right)}{dr} \right\vert_{r=R}=-\frac{\sigma_{q}}{\varepsilon_{o}\varepsilon}$  $\lim_{r\to\infty} \psi\left( r \right)=0$ | (5) |
| --- | --- | --- | --- |

Equations 3 through 5 and the incompressibility equation, which is explicitly imposed at all positions, constitute a system of equations that are solved numerically. To accomplish this, these equations are converted from those that are continuous in space to their discretized counterparts. Specifically, space is discretized to spherical shells with spacing of 0.2 nm.

**References**

1. Gole A, Murphy CJ: Polyelectrolyte-coated gold nanorods: Synthesis, characterization and immobilization. *Chem Mater.* 2005;17(c):1325–1330.

2. Davis TJ, Soto-Ortega DD, Kotarek JA, Gonzalez-Velasquez FJ, Sivakumar K, Wu L, Wang Q, Moss MA: Comparative study of inhibition at multiple stages of amyloid-β self-assembly provides mechanistic insight. *Mol Pharmacol.* 2009;76:405–413.

3. Kotarek J A., Moss M A.: Impact of phospholipid bilayer saturation on amyloid-β protein aggregation intermediate growth: A quartz crystal microbalance analysis. *Anal Biochem.* 2010;399:30–38.

4. McQuarrie DA: *Statistical Mechanics*. Mill Valley, California: University Science Books; 2000.

5. Carignano M A., Szleifer I: Structural and thermodynamic properties of end-grafted polymers on curved surfaces. *J Chem Phys.* 1995;102:8662.

6. Raphael E, Joanny J-F: Annealed and Quenched Polyelectrolytes. *Europhys Lett.* 1990;13:623–628.

7. Davis HT: *Statistical Mechanics of Phases, Interfaces, and Thin Films*. New York, New York: Wilet-VCH; 1996.
